# KSHV/HHV-8 and HIV infection in Kaposi's sarcoma development

**DOI:** 10.1186/1750-9378-2-4

**Published:** 2007-02-02

**Authors:** Pawan Pyakurel, Fatemeh Pak, Amos R Mwakigonja, Ephata Kaaya, Peter Biberfeld

**Affiliations:** 1 Immunopathology Lab., Department of Pathology and Oncology, Karolinska Institutet, 171-76 Solna, Stockholm, Sweden; 2 Muhimbili University College of Health Sciences, P. O. Box 65023, Dar-Es-Salaam, Tanzania

## Abstract

Kaposi's sarcoma (KS) is a highly and abnormally vascularized tumor-like lesion affecting the skin, lymphnodes and viscera, which develops from early inflammatory stages of patch/plaque to late, nodular tumors composed predominant of spindle cells (SC). These SC are infected with the Kaposi's sarcoma-associated herpesvirus or human herpesvirus-8 (KSHV/HHV-8). KS is promoted during HIV infection by various angiogenic and pro-inflammatory factors including HIV-Tat. The latency associated nuclear antigen type 1 (LANA-1) protein is well expressed in SC, highly immunogenic and considered important in the generation and maintenance of HHV-8 associated malignancies. Various studies favour an endothelial origin of the KS SC, expressing "mixed" lymphatic and vascular endothelial cell markers, possibly representing hybrid phenotypes of endothelial cells (EC). A significant number of SC during KS development are apparently not HHV8 infected, which heterogeneity in viral permissiveness may indicate that non-infected SC may continuously be recruited in to the lesion from progenitor cells and locally triggered to develop permissiveness to HHV8 infection. In the present study various aspects of KS pathogenesis are discussed, focusing on the histopathological as well as cytogenetic and molecular genetic changes in KS.

## Background

### Kaposi's sarcoma

Kaposi's sarcoma first described by Moritz Kaposi in 1872 as "idiopathic multiple pigmented sarcomas of the skin" [1] is an angioproliferative, tumour-like lesion usually developing in the skin [2], and eventually disseminating to multiple cutaneous sites, viscera and lymph nodes. Previously a rare disease, it is now a global health care and clinical problem because of its association with the HIV pandemic [3] and other immunosuppressed states[4].

Four clinically different KS forms are now recognized [5]:

a) Classical or sporadic KS (CKS), originally described [1] as a slow growing, indolent tumor mostly developing in the extremities of elderly males of eastern and Mediterranean Europe. b) Endemic KS (EKS), predominant in eastern and central sub-Saharan Africa before the AIDS epidemic and clinically similar to CKS, but also seen in a more fulminant and fatal form in children. The childhood EKS is often lymphoglandular with or without skin involvement. c) Acquired immunodeficiency syndrome (AIDS)-associated KS (AKS), the most frequent tumor of human immunodeficiency virus type I (HIV-l) infection and the most aggressive and rapidly growing form of KS in AIDS, with early dissemination in the skin and viscera.

d) Iatrogenic KS (IKS), seen in drug related immunosuppressed patients, e.g. transplant patients, emphasizing the importance of immune disturbance as a co-factor in the pathogenesis of IKS and AKS, and possibly also EKS.

In spite of the clear clinical differences the histopathology of the various KS forms is essentially the same, with characteristic changes related to stage in the development of the KS tumor[6].

The epidemiology of AKS led to the discovery of a novel herpes virus [7], which subsequently was shown to be associated with all clinico-epidemiological forms of KS [8]. The virus was rapidly characterised as a KS associated herpes virus (KSHV) and classified as human herpes virus type 8 (HHV-8). It was soon recognized to also be associated with some rare types of lymphomas in AIDS patients, namely primary effusion lymphoma or body-cavity-based-lymphoma (PEL/BCBL) and Castleman's disease (MCD)[9].

### Human herpesvirus type 8 (HHV-8)

Human herpesvirus 8 or Kaposi's sarcoma associated herpesvirus (HHV-8/KSHV) was recognized to be a novel gamma-2 herpesvirus of the rhadinovirus genus closely related to the human gamma -1 herpesvirus, Epstein-Barr virus (EBV) [10].

A number of viral glycoproteins have been characterized shown to bind to cell surface heparan sulfate [11] and the cell receptor integrin α3β1, respectively, thereby mediating virus entry through endocytosis [12]. In the KS lesion HHV-8 is predominantly found in the so called tumor spindle (SC) cells in KS but was also in some lymphocytes, monocytes and keratinocytes [13]. The virus replicates in either a lytic or predominantly in the latent form as closed circular episomal DNA [14] within the nucleus of KS tumor cells (SC) and B cells of MCD and other infected mononuclear cells [15]. It has been shown that the episomal viral DNA is tethered to metaphase chromosomes and copied in tandem with host cell DNA during cell division [16]. Latent viral specific genes well demonstrated in infected KS SC are the latent nuclear antigen (LANA-1), viral cyclin (v-cyclin), v-FLIP and kaposin a small membrane protein, which are all adjacent in the genome [16]. Lytic virus expression is most frequent in MCD, moderate in KS and relatively rare in PEL cells. Common viral genes found during lytic expression include K1 transmembrane protein, v-GCR, v-IRF, v-IL-6 and v-MIP [15].

### LANA-1

**Figure 1:**
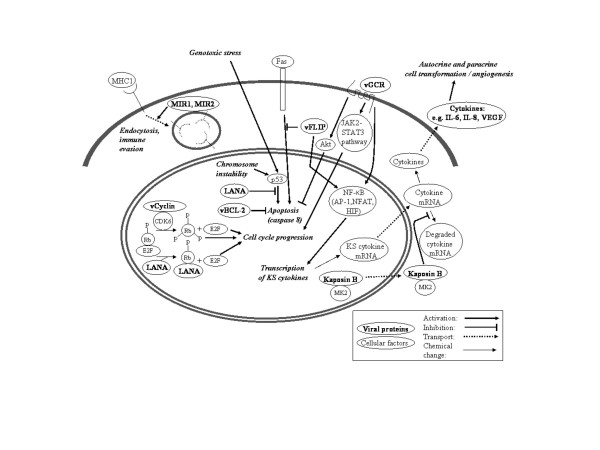
HHV-8 gene expression (pathogenesis) during SC development and tumor growth.

The latency associated nuclear antigen type 1 (LANA-1) protein is a well expressed and highly immunogenic, latent nuclear antigen of HHV-8 considered important in the generation and maintenance of HHV-8 associated malignancies [17] by its cell cycle regulation in competing with E2F for binding of hypophosphorylated pRb thus freeing E2F to activate gene transcription involved in cell cycle progression [18] (Fig 1). E2F activity can also trigger apoptosis via the p53 pathway but LANA-1 interacts with p53, repressing its gene transcriptional activity and ability to induce apoptosis (Fig 1). Therefore the inhibition of p53 by LANA-1 allows latent HHV-8 to promote cell cycle progression whilst inhibiting apoptosis [19]. Oncogenic viruses often block cell differentiation during tumor development by the stabilization of beta-catenin which also appears to be promoted by LANA [20].

The LANA-1 antigen is well detectable by immunohistochemistry also in routinely formalin fixed paraffin embedded biopsies. It is expressed by most SC in both early and late stage lesions of all different clinical KS forms (AKS, EKS, CKS and IKS) [8,21] and therefore used as a diagnostic marker in suspected HHV-8 related lesions and also for serology of LANA-1 antibodies in patients by immunocytochemistry that gives a characteristic speckled nuclear staining on HHV-8 infected BCBL cells. Several studies have shown an increase in LANA-1 positive cells during progression of KS lesions [22,23] allowing quantification and phenotyping of these cells in KS lesions.

### Pathogenesis of KS

HHV-8 is the most recently identified human *oncogenic *herpesvirus [24] expressing candidate viral oncogenes which constitutively activate growth-signalling pathways [13,25]. The pathogenesis of KS is however still unclear and appears complex, involving various mechanisms dependent on both viral and cellular activities related to inflammation and angiogenesis promoted by endothelial growth factors (β-FGF, PDGF, VEGF) including HIV-Tat as well as cell proliferation and anti-apoptosis (vBCL2) [2,13,26,27]. Characteristic for HHV-8 is the high homology of several viral and cellular genes suggesting viral genes were pirated from host chromosomes during viral evolution. Some of these genes are involved in down modulating the host immune responsiveness to target, infected cells and modulate cell proliferation, cell differentiation and angiogenesis [13], including genes as vBcl-2, vIL-8R, vMIPs, vIL-6, and the D type viral cyclin

The HHV-8 infected cells escape immune response targeting by down regulation of surface MHC mediated by two transmembrane proteins, MIR1 and MIR2 [28] (Fig 1), which promote MHC endocytosis, and lysosomal degradation (Fig 1). Downregulation of MHC I and its accessory immune receptors poses the risk of initiating a natural killer (NK) cell response by initiating apoptosis through Fas (CD95/Apo-1) in cells lacking appropriate MHC I expression. However, HHV-8 can inhibit NK-mediated killing through expression of the anti apoptotic v-FLICE-inhibitory proteins (v-FLIPs) [28]. V-FLIP which acts as a dominant inhibitor of receptor-activated apoptosis by binding to Fas-associated death domain protein and caspase 8 (FLICE) [29]. This prevents activated caspase recruitment into the death-inducing signaling complex (Fig 1).

HHV-8 v-FLIP shares with c-FLIPs the ability to activate NF-κB [28] which is essential for the growth and survival of the cell. Our studies on KS biopsies have shown that apoptosis clearly decreases during development of early to late nodular KS lesions [30], and that the expression of anti-apoptotic v-FLIP and cellular Bcl-2 increase from early to late stage KS lesions, [30,31]. Thus viral exploitation of these two anti-apoptotic pathways contributes to the tumor-like growth and progression of the KS lesion.

V-cyclin binds with cyclin dependent kinases (CDK6), which complex phosphorylates pRb, releasing a transcription factor (E2F), which activates the transcription of S-phase genes (Fig 1). However, unlike cellular cyclin, vCyclin-CDK6 complexes are resistant to CDK inhibitory proteins, which may lead to unregulated cell cycle progression and transformation and thereby promote tumor development [32].

Kaposin, the latency gene represents a potential viral oncogene and is characterized as a transforming gene [33], although little is known about its role in deregulating cell signalling [34]. It is present in three (A, B, C) isoforms [35] of which Kaposin B is expressed by all HHV-8 infected cells and can activate the p38-MK2 pathway [36] (Fig 1) and block the degradation of the messenger RNAs transcribing various cytokines necessary for cell survival, hence increasing their translation [36]. The Kaposin gene also encodes several microRNAs (miRNA), which may regulate gene expression by binding to complementary messenger RNAs [37]. Two of these HHV-8 miRNAs are expressed by SC at all KS stages [37] and may contribute to tumorigenic transformation of infected cells [33], and therefore of therapeutic interest [37].

The KSHV miRNAs are expressed from what appears to be a single genetic locus that largely coincides with an 4-kb noncoding sequence located between the KSHV *v-cyclin *and *K12 Kaposin *genes, both of which are also expressed in latently infected cells. Computer analysis of potential mRNA targets for these viral miRNAs identified a number of interesting candidate genes, including several mRNAs previously shown to be downregulated in KSHV-infected cells. It appears that these viral miRNAs play a critical role in the establishment and or maintenance of KSHV latent infection and hence, in KSHV-induced oncogenesis [38].

HHV-8 also encodes a G-protein-coupled receptor (vGCR) homolog to the human angiogenic, chemokine interleukin-8 receptor (IL-8R, CCR1 and CXCR2) [39] (Fig 1). Angiogenic responses induced by vGCR are mediated by upregulation of vascular endothelial growth factor (VEGF) [40]. The constitutive activity of vGCR could therefore have a role for VEGF expression by SC during the development of early stage KS lesions [41]. Furthermore the vGCR dependent expression of autocrine and paracrine growth factors (bFGF, VEGF,) promotes the angiogenesis and edema [26,42] seen in KS patients. It was also shown [43] that viral envelop glycoprotein gB can activate the VEGFR-3 receptor and trigger receptor signalling on the surface of microvascular endothelial cells, thereby modulating cell migration and proliferation. VEGFR-3 expression and activation may also enhance HHV-8 infection and participate in HHV-8 mediated transformation [43] and thereby appears to be an important factor in the pathogenesis of Kaposi's sarcoma.

The Kaposi sarcoma herpesvirus (KSHV) also encodes multiple proteins that disrupt host antiviral responses, including four viral proteins that have homology to the interferon regulatory factor (IRF) family of transcription factors. At least three of the KSHV vIRFs (vIRFs 1–3) alter responses to cellular IRFs and to interferons (IFNs). The vIRFs also affect other important regulatory proteins in the cell, including responses to transforming growth factor beta (TGF-beta) and the tumor suppressor protein p53 [44]. K7/vIAP (inhibitor of apoptosis protein) is another antiapoptotic factor homologous to the cellular protein survivin [45].

### Histogenesis of KS

The histopathology of KS is characterized by an early infiltration of mononuclear inflammatory cells, formation of small, irregular, endothelial lined slits around new blood vessels (angiogenesis) and extravasation of erythrocytes [2] with accumulation of hemosiderin pigments. KS at early stages appears to reflect a predominantly reactive cell proliferation of polyclonal nature that may regress, but usually progresses to a nodular possibly clonal tumor [2,46]. Pathognomonic for KS development from early patch/plaque to late nodular tumor lesions is the increased appearance of bundles of morphological spindle cells (SC) expressing CD34 (hematopoietic stem cell and vascular endothelial marker). At the late nodular KS stage there is less inflammatory cell infiltration, mostly around the boaders of the dense, nodular accumulation of SC bundles which skin lesions may later ulcerate. Unlike typical metastatic cancers, KS often appears early as a multicentric tumour, with each lesion arising de novo by a localized small patch and of SC [47].

Most SC are positive for CD34 and LANA but a considerable number of CD34+ SC are LANA- at all AKS/EKS stages [22,23]. This apparent heterogeneity in viral permissiveness of CD34+ SC seems less compatible with a clonal CD34+ SC proliferation and virus transfer but appears to indicate that also non-infected CD34^+ ^SC are continuously recruited from progenitor cells and locally triggered to develop permissiveness to HHV-8 infection [22,23]. Furthermore cells belonging to the non-cycling SC (Ki67-) population showed a clear increase during development from patch/plaque (median 13.5%) to nodular stage (median 40.3%) [22], also supporting the concept of continuous recruitment of CD34+ cells to the lesion. KS spindle-like cells have been shown to develop in cultures of peripheral blood of HIV infected patients with KS or at high risk for developing KS [48]. Furthermore recent studies show that endothelial cells or their precursors residing in donor kidneys may contribute to post-renal transplant KS indicated by the finding that KS SC in the female recipient kidney had a male (donor) karyotype and that KS SC expressed the donor HLA antigen [49]. These findings appear to indicate that KS SC and/or their progenitors can be recruited during development of KS lesion.

Characteristic spindle cells (SC) express various "mixed" (LEC and VEC) endothelial phenotypic cell markers possibly representing hybrid phenotypes of endothelial cells at different maturation stages. It has been recurrently debated whether SC are vascular (VEC) or lymphatic (LEC) in origin or derive from mesenchymal progenitor cells [50-52], although most studies by immunohistochemistry have revealed that SC express lymphatic markers, such as D2-40 [53], LYVE-1 [50] and VEGFR-3 [51]. Also studies by gene expression microarray show that KS neoplastic cells are closely related to lymphatic endothelial cells (LEC) but coexpressing some blood vascular endothelial cell (VEC) markers [54]. Furthermore HHV-8 can infect both LEC and VEC in vitro and infected LEC had a higher HHV-8 genome copy number than VEC[54]. In-vitro infection of CD34+ human dermal microvascular endothelial cells (HDMEC) with HHV-8 resulted in the upregulation of LEC markers such as LYVE-1 in the infected HDMEC [55].

In our study all LANA+ cells were LYVE-1+ (lymphatic endothelial markers) in early and late KS and the HHV-8 infection (LANA) appeared better correlated to LYVE-1 than to CD34 expression [23]. LANA+/CD34-cells were more frequent in early as compared to late lesions and did not express a leucocytic phenotype (CD3, CD20, CD45, CD68) [22], but most expressed lymphatic endothelial (LEC) markers such as LYVE-1, VEGFR-3 and D2-40, suggesting that resident LECs represent an early target of primary HHV-8 infection [23]. This is also supported by other findings [54] that infected LECs have a higher HHV-8 genome copy number than VECs. Obviously a high viral copy number may result in an efficient maintenance and propagation of episomal HHV-8 DNA in dividing and migrating LECs. Furthermore in-vitro activation of VEGFR-3 by HHV-8 has been shown to increase endothelial cell migration and to enhance cell susceptibility to HHV-8 infection and transformation [43]. Hence, the activation of VEGFR-3 in LANA+/VEGFR-3+ SC observed during KS development will probably promote an increased endothelial cell migration (recruitment) and transformation to tumor SC including formation of pathological vascular slits.

Cell proliferation is relatively low in KS as shown by our previous studies on proliferation related protein Ki67 expression and DNA flow cytometry [30]. The frequency of proliferating (Ki67+) cells usually decreased during development from early to late KS lesions, consistent with the notion that KS growth from a early reactive lesion to a nodular tumor depends not only on SC division but also on decreased apoptosis [30] and progenitor recruitment [22,23]. No significant difference in cell proliferation was observed between nodular AKS and EKS [22]. These findings could therefore indicate that the usually more spread and aggressive growth of the AKS tumors may reflect a higher rate of SC progenitor recruitment compared to the more indolent EKS lesions.

### Cytogenesis of KS

Reports on cytogenetic and molecular genetic changes in KS are few [56]. Studies from KS cell lines, KS Y-1 (AKS derived) and KS SLK (IKS derived) revealed loss of copies of chromosomes 14 and 21 and non-random translocations and deletions in the short arm of chromosome 3 at region 3p14. These KS cell lines also exhibit loss of heterozygosity of loci at region 3p14-ter. The chromosome 3 alterations observed were suggested to contribute to the neoplastic process in KS [57] but other cytogenetic studies on the KS-IMM cell line (IKS) [56] showed gains in 1q10→qter, 7p10→pter, 7q22→qter, 8p11→qter, 14pter→q22 but no changes in chromosome 3 [56]. These aberrations are compatible with the notion that initially KS may develop as a reactive polyclonal cell proliferation associated with chromosome instability, followed by acquisition of clonal chromosome changes in later stages [56]. However, the significance in KS pathogenesis of aberrations on established KS cell lines should be related to finding that such cell lines when established usually loose their HHV-8 episomes and possibly may represent only a minor KS cell population.

The chromosomal instability suggested by the studies on cell lines may lead to cell apoptotis via the p53 pathway[58]. However, HHV-8 LANA binds to p53repressing its ability to induce apoptosis [59]. Furthermore telomerase activity has been found to be upregulated in KS [60], which may immortalise the infected cell leading to increased tumor cell survival.

Previous, CGH studies of formalin fixed paraffin embedded KS biopsies revealed a recurrent gain at 11q13 [61], which also amplifies two known oncogenes, FGF4 and INT2, residing at 11q13 suggesting a possible role of HHV-8 in the amplification and activation of genomic oncogenes [61].

Loss of chromosome Y was observed in most AKS and EKS cases recently studied by us [62] and interestingly it was the only aberration observed in early KS. Late stage (nodular) KS had beside loss of chromosome Y, also recurrent deletions on chromosomes 16 and 17. Deletion of chromosome Y was also reported by previous studies on short term cultures of primary KS tumor cells and established KS cell lines [56]. EKS showed often more chromosomal abnormalities than AKS [62], which might indicate that genomic instability could be a more important factor in the development of EKS than AKS. Most likely AKS development is also promoted by various cytokines and growth factors produced by the HIV infection and the dysregulated and compromised state of host immune response. Loss of the Y chromosome and encoded male specific minor histocompatibility antigens (HY antigen) has been shown to be linked to haematological relapse in acute lymphoblastic leukemia due to immune escape mechanisms [63]. The HY antigens are presented at the cell surface with the major histocompatibility complex (MHC) and together also processed intracellularly [64]. However, no studies have previously indicated a deficiency of HY antigen in KS tumors, which loss of the Y chromosome in our studies suggests as of possible importance in KS pathogenesis.

### HIV pathogenesis in KS

There seems to be a cross talk between HIV-1 and HHV-8 as recent studies have shown that HIV-1 replication stimulates HHV-8 production in PEL cell lines and peripheral blood mononuclear cells from KS patients, possibly due to the activating functions of HIV-Tat [65, 66] ORF50, the major transactivator of HHV-8 lytic cycle can also induce increased levels of HIV replication by interacting synergistically with HIV-1 Tat leading to increased cell susceptibility to HIV infection and transient permissiveness to HIV replication [67].

The increased incidence of KS in patients with AIDS was also shown to be related to effects of the HIV-1 Tat protein by stimulation of proliferation and anti-apoptosis of infected spindle cells (SC) and also activation of HHV-8 thus increasing SC viral load and expression of various viral genes with oncogenic potential (vGCR, vBCL2, and vIRF1, see above) [65]. Thus, Tat promotes tumorigenesis of endothelial cells, both via stimulation of vascular endothelial growth factors, anti-apoptotic activity and HHV-8 replication. Notably, the functional activity of Tat protein in the pathogenesis of AKS clearly involves an intercellular signalling cascade which is inhibited by antibodies to HIV-Tat epitopes [68, 69].

Recently we have found differences in SC viral load between oral and cutaneous KS lesion also suggesting possible differences of Tat expression in these lesions [70].

In summary the concept of oncogenesis related to infection is particularly well exemplified by the herpes virus HHV-8 and retrovirus HIV-1 associated Kaposi's sarcoma, which develops due to the effects of various host-cells and viral factors elicited during infection affecting cell proliferation, cell escape from apoptosis and dysregulation of host immune responses.
